# Transperitoneal Subcostal Access for Urologic Laparoscopy: Experience of a Large Chinese Center

**DOI:** 10.1155/2016/4062390

**Published:** 2016-12-15

**Authors:** Lei Zhang, Dong Fang, Xuesong Li, Lin Yao, Gengyan Xiong, Zhisong He, Liqun Zhou

**Affiliations:** Department of Urology, Peking University First Hospital, Institute of Urology, Peking University, National Urological Cancer Center, Beijing 100034, China

## Abstract

*Objective*. To present our experience of using transperitoneal subcostal access, Palmer's point (3 cm below the left costal margin in the midclavicular line), and its right corresponding site, in urologic laparoscopy.* Methods*. We used Palmer's point and the right corresponding site for initial access in 302 urologic surgeries (62 cases with prior surgeries). The record of these cases was reviewed.* Results*. Success rate of initial access is 99.4%, and complication rate of puncturing is only 3.4% with no serious complication. In the cases with prior surgeries, there were only two cases with access complication on the right side (minor laceration of liver). For people with BMI more than 30 kg/m^2^ (12, 3.9%), the success rate was also 100 percent.* Conclusions*. Palmer's point and the corresponding right location are feasible, effective, and safe for initial access in urologic laparoscopic surgeries. This entry technique should be used routinely in urologic laparoscopic surgeries.

## 1. Introduction

The establishment of pneumoperitoneum is considered to be a dangerous step in laparoscopic surgery. Potential complications include injury of vessels, the gastrointestinal tract, and the urinary tract as well as gas embolism. Approximately 50% of all laparoscopic complications have been attributed to the entry technique [1]. The risk is higher for patients with a history of abdominal surgery because of adhesion.

In 1974, Palmer [2] first described an abdominal entry point for patients with prior surgery located 3 cm below the left costal margin in the midclavicular line. This entry point, now known as Palmer's point, is noted to produce good results when establishing pneumoperitoneum [3–10]. However, all of the published guidelines have restricted their recommendations for Palmer's point to patients in whom adhesions are suspected [11, 12]. The value of Palmer's point may therefore be underestimated.

There are only a few published studies concerning the use of Palmer's point and the corresponding right point as the initial access sites in laparoscopic surgeries [3–10]. In these studies, the points were primarily used for gynecologic operations [3–6, 8, 10]; only two articles describe urological surgeries [7, 9]. No studies have described the use of Palmer's point as the initial access site for Chinese patients.

We used transperitoneal subcostal access (Palmer's point and its corresponding right point) for establishing pneumoperitoneum in 302 urologic laparoscopic surgeries. To the best of our knowledge, this study is the first report of transperitoneal subcostal access in urologic laparoscopy for Chinese patients.

## 2. Materials and Methods

A retrospective review of studies describing laparoscopic surgeries using Palmer's point or the corresponding right point to establish pneumoperitoneum between April 2009 and September 2013 was conducted. Palmer's point or the corresponding right point was used for left- or right-sided operations, respectively. A total of 302 cases were identified (Table 1), including radical nephrectomy (*n* = 131; 43.4%), nephroureterectomy (*n* = 78; 25.8%), partial nephrectomy (*n* = 32; 10.6%), pyeloplasty (*n* = 29; 9.6%), simple nephrectomy (*n* = 12; 4.0%), adrenalectomy (*n* = 10; 3.3%), renal cyst decortication (*n* = 6; 2.0%), and segmental ureterectomy (*n* = 4; 1.3%). The patient demographics are shown in Table 1. The average age was 55.01 ± 16.90 years (range, 16 to 88 years). The average BMI was 24.01 ± 3.16 kg/m^2^ (range, 16.53–35.22 kg/m^2^), 12 patients were more than 30 kg/m^2^, 22 patients were less than 20 kg/m^2^, and 268 patients were between 20 kg/m^2^ and 30 kg/m^2^. Forty-six patients were ASA I, 227 patients were ASA II, and 29 patients were ASA III. Sixty-two (20.5%) cases were with prior abdominal surgery history. Previous surgical history and patients habitus were not contraindications to blind insertion of the Veress needle.

**Figure 1:**
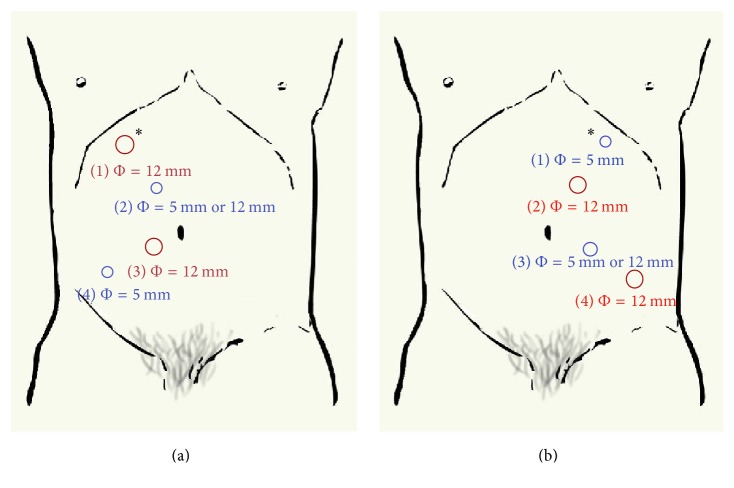
Transperitoneal subcostal access for urologic laparoscopy. (a) Trocar site for right-sided operations. (b) Trocar site for left-sided operations. ^*∗*^Primary insertion site.

Under general anesthesia, the patient is placed in the lateral recumbent position (45 degrees from horizontal) with the lesion side up. For the left-sided operation, a 1 cm horizontal incision was made 3 cm below the left costal margin in the midclavicular line (Palmer's point) (Figure 1(b)). The Veress needle was held similar to a dart to perpendicularly penetrate the skin using the dominant hand. The procedure of penetrating the skin should be slow, and the breakthrough should be felt two or three times before entry into the cavity. A saline drop test was used to confirm the entry into the peritoneal cavity. Carbon dioxide was insufflated into the cavity at a low flow rate, and the intra-abdominal pressure was shown on the monitor. The initial pressure was usually 4-5 mmHg. If the pressure increased slowly from it to 14 mmHg, pneumoperitoneum was successfully established. Otherwise, the carbon dioxide may not enter into the cavity or the flow was obstructed, and the needle should be removed. A semi-open entry was used after attempting the procedure more than three times. Once pneumoperitoneum was established, a trocar (10 mm) was inserted through the initial puncture site. A 30° laparoscope was inserted through the trocar to identify whether the viscera or vessels were injured. Other trocars were inserted using the aid of the laparoscope. For right-sided operations, the primary puncture site was the corresponding site of Palmer's point, 3 cm below the right costal margin in the midclavicular line. The subsequent procedure was commensurate with that of the left side.

The complications relative to the blind insertion of the Veress needle were recorded, including minor and major complications. The minor complications were defined as those that did not affect the length of hospital stay. The major complications included those leading to death, those requiring conversion to an open operation, and those requiring prolonged hospitalization.

## 3. Results and Discussion

The layout of the trocar is shown in Figures 1(a) and 1(b) for right- and left-sided operations, respectively. Of the 302 transperitoneal laparoscopic operations, the Veress needle was successfully inserted in 299 cases (99.0%). Three failed cases were not associated with prior abdominal surgeries, obesity, or thinness. No open operation was performed because of puncturing complications. No serious complications occurred, such as perforation of vessels or the gastrointestinal tract. Minor complications occurred in ten cases (3.3%): five cases demonstrated injury of Glisson's capsule, three cases demonstrated injury of the omentum, and two cases demonstrated injury of the falciform ligament. After a simple hemostasis, the operations continued, and there were no sequelae. In all 62 cases with previous surgery, adhesions of different degrees were detected. However, the Veress needle insertions were successful in all of these cases, and there were only four cases with access complications (minor injury of Glisson's capsule). For individuals with BMIs > 30 kg/m^2^ (*n* = 12; 3.9%) and <20 kg/m^2^ (*n* = 22; 7.3%), the success rate was 100 percent. After establishing pneumoperitoneum, the initial access site could be used for the insertion of the laparoscope and other instruments. No incision infection or pneumothorax was found postoperatively. None of the patients developed an incision hernia at the trocar site after a 28-month follow-up.

Entry into the peritoneal cavity and the establishment of pneumoperitoneum constitute the most dangerous steps in laparoscopic surgery; approximately 50% of all complications occur during these steps [1]. Several complications, such as gastrointestinal tract perforation and massive hemorrhage, could result in fatal outcomes. Recently, many methods have been proposed for minimizing injuries, especially in patients with prior surgeries and intraperitoneal adhesions. There is no consensus on which approach is the safest [13].

The periumbilical region is the traditional site of initial access in laparoscopic surgery because the abdominal wall is thin, which facilitates insertion of the needle. However, there are several problems concerning this access technique. First, the periumbilical region is always unclean and more susceptible to infection. There are several great vessels under the umbilicus. When instruments are inserted in the periumbilical region, the risk of major vessel injury is between 0.5 and 6.4 per 100 laparoscopies [14–16], which could be lethal. Furthermore, the patient's weight has a considerable effect on the periumbilical abdominal wall thickness. The strength required for inserting the Veress needle is markedly different between thin and obese patients. Moreover, for patients with prior abdominal surgery, there is a two-fold increased risk of access complication at the umbilical site [17]. Thus, the use of this technique is restricted, especially for patients with prior surgery.

In 1974, Palmer first described a puncture site, now known as Palmer's point that could be used in patients with prior surgeries [2]. Previous studies have shown satisfactory results for establishing pneumoperitoneum at Palmer's point or the corresponding right point (Table 2). The success rate for establishing pneumoperitoneum at these points was 93–100%, although a significant portion of the patients had prior surgeries. However, few studies have described the use of Palmer's point for establishing pneumoperitoneum, and no studies have been performed on Asian patients. This is likely because all the guidelines have restricted their recommendations for using Palmer's point to cases in which abdominal adhesions are suspected. Moreover, the majority of laparoscopic operations have been performed by gynecologic surgeons for the treatment of pelvic lesions. Periumbilical insufflation is traditionally used by gynecologists, and there are no prospective studies that document the safest entry technique. Hence, the surgeon's choice is usually based on this practice.

Based on the satisfactory results achieved with this technique, we believe that the value of Palmer's point for Western patients is underestimated. However, the habitus differs between Western and Chinese patients. The value of Palmer's point and its corresponding point as the initial access points for Chinese people is unknown in urologic surgeries of the upper urinary tract. We expanded the use of Palmer's point without restriction for patients with suspected adhesions. Finally, we used Palmer's point and the corresponding right point for primary needle insertion in 302 transperitoneal laparoscopic operations. The results were compelling, with a success rate of 99.0%, a complication rate of only 3.3%, and no serious complications. However, we should note that Palmer's point corresponding site on the right side is not as safe as Palmer's point because there are liver, gallbladder, duodenum, and vena cava under the right site. It is reasonable to move the right entrance site about 2 cm below comparing to the left side.

The use of Palmer's point or the corresponding right point for establishing pneumoperitoneum has several advantages. First, because the peritoneum is fixed and braced anteriorly by the arch rib, inserting the Veress needle requires less effort; thus, abdominal traction (e.g., using two towel clips) is unnecessary. Second, there is less subcutaneous fat at Palmer's point, even in obese patients [18]. Thus, inserting the needle is easy and is unaffected by the shape of the patient. Third, with the aid of gravity, the viscera fall away from Palmer's point. There are no major vessels at this site. Inserting the Veress needle in this area is theoretically safe. Additionally, the average distance from Palmer's point to the aorta is 11.3 ± 0.2 cm [18]. When inserting the Veress needle caudally at 45°, the distance is extended to 16.6 ± 0.2 cm [18]. Although the length of the Veress needle is usually 12 cm, the risk of aortic injury approaches zero. Third, adhesion is rare in these areas, even in patients with prior surgeries.

Based on our experience, transperitoneal subcostal access is feasible, effective, and safe in urologic laparoscopic surgeries. Consistent with our opinion, Chung et al. [7] have proposed that this access technique can be routinely used in transperitoneal laparoscopic surgery, especially that involving the upper urinary tract, and should not be reserved for patients with suspected adhesions. Additionally, Tüfek et al. [9] indicated that Palmer's point Veress needle access was a safe and effective method for establishing pneumoperitoneum in patients subjected to robotic and standard laparoscopic radical prostatectomy.

Our experience showed that the success rate for establishing pneumoperitoneum was 100% in patients with BMIs > 30 kg/m^2^ and <20 kg/m^2^, which indicated that the use of transperitoneal subcostal access was unaffected by the patient's shape. Approximately 50% of the complications were attributed to injury of Glisson's capsule in this series. We should apply caution when using these locations as the initial access point for patients with large livers. Under these circumstances, the puncture site should be moved downward to avoid the margin of the liver.

This study had several limitations. First, it was a retrospective study conducted in a single institution with selection bias. Second, all the puncturing procedures were performed by a single surgeon with considerable experience. These compelling results may not be reproduced by a surgeon with less experience. With increased experience, the use of this technique could produce satisfactory outcomes. Third, because of the retrospective nature of this study, it is still uncertain whether transperitoneal subcostal access is better than periumbilical access for initial access in laparoscopic surgeries. Prospective randomized controlled trials are warranted.

## 4. Conclusions

In summary, our initial experience demonstrated that Palmer's point or the corresponding right point was feasible, effective, and safe for initial access in urologic laparoscopic surgeries. These sites should be used routinely for establishing pneumoperitoneum in urologic laparoscopic surgeries and should not be limited to patients with suspected adhesions.

## Figures and Tables

**Table 1:** Patient demographics.

Sex, *n* (%)	
Women (%)	142 (47.0%)
Men (%)	160 (53.0%)
Age, years	
Mean ± SD	55.01 ± 16.90
Median, range	57, 16–88
BMI, kg/m^2^	
Mean ± SD	24.01 ± 3.16
Median, range	23.99, 16.53–35.22
Side, *n* (%)	
Right	136 (45.0%)
Left	164 (54.3%)
Bilateral	2 (0.7%)
Surgery history, *n* (%)	
Presence	62 (20.5%)
Absence	240 (49.5%)
Duration of follow-up, months	
Mean ± SD	28.0 ± 11.59
Median, range	28.0, 7–60

**Table 2:** Review of Palmer's point or the corresponding right point for initial access in laparoscopic surgeries.

Reference	Year	Number of cases	Success rate of puncturing	Rate of complication	Rate of conversion to open operation
Childers et al. [3]	1993	41	97.6%, 40	2.4%	2.4%
Chang et al. [4]	1994	17	100%	0	17.6% (3), serious adhesion
Parker et al. [5]	1999	17	100%	0	0
Patsner [6]	1999	90	100%	2.2%	1.1%
Chung et al. [7]	2003	622	93%	8% (minor complications)	0
Tulikangas et al. [8]	2003	267	98.5%, 263	1.12%	0
Tüfek et al. [9]	2010	147	100%	0	0
Granata et al. [10]	2010	136	98.5%	0	0
